# Effect of Shin'iseihaito (Xinyiqingfeitang) on Acute* Streptococcus pneumoniae* Murine Sinusitis via Macrophage Activation

**DOI:** 10.1155/2017/4293291

**Published:** 2017-07-20

**Authors:** Masaaki Minami, Toru Konishi, Hiroshi Takase, Zhixia Jiang, Tetsuya Arai, Toshiaki Makino

**Affiliations:** ^1^Department of Bacteriology, Graduate School of Medical Sciences, Nagoya City University, 1 Kawasumi, Mizuho-ku, Nagoya, Japan; ^2^Department of Pharmacognosy, Graduate School of Pharmaceutical Sciences, Nagoya City University, 3-1 Tanabe-dori, Mizuho-ku, Nagoya, Japan; ^3^Core Laboratory, Graduate School of Medical Sciences, Nagoya City University, 1 Kawasumi, Mizuho-ku, Nagoya, Japan; ^4^R&D Center, Kobayashi Pharmaceutical Co., Ltd., 4-10 Doshomachi 4-chome, Chuo-ku, Osaka, Japan

## Abstract

*Streptococcus pneumoniae (S. pneumoniae)* causes sinusitis. The general treatment of* S. pneumonia *sinusitis is by using antibiotics; however, one of their serious problems is the attenuation of their effect. Shin'iseihaito (Xinyiqingfeitang), a formula of Japanese traditional Kampo medicine, has been used for the treatment of sinusitis in Japan. In this study, we investigated the efficacy of Shin'iseihaito against* S. pneumoniae*-caused sinusitis in mice. Oral administration of Shin'iseihaito extract (SSHT) decreased the nasal colonization of* S. pneumoniae *in both prophylactic and therapeutic treatments, respectively, and the former was more effective than the latter. Histopathological analysis revealed that the epithelial tissue from* S. pneumoniae*-infected nose under SSHT treatment recovered the tissue destruction in comparison to infected nose. We also confirmed this result by scanning electron microscopic analysis. Murine peritoneal macrophages from SSHT-treated mice had significant phagocytic activity in comparison to those from untreated group. We also found that tumor necrosis factor-*α*, interleukin-1*β*, interleukin-6, and monocyte chemotactic protein-1 levels and the migration of macrophages from* S. pneumoniae*-infected mice with the treatment with SSHT were increased compared to those from untreated group. Our data suggest that Shin'iseihaito may be useful for the treatment of* S. pneumoniae*-induced sinusitis.

## 1. Introduction

*Streptococcus pneumoniae (S. pneumoniae)* is the gram-positive bacterium which is the most common pathogen causing sinusitis, otitis media, pneumonia, meningitis, and sepsis, especially in young children and the elderly [1]. Although antibiotics such as penicillin have been effective against* S. pneumoniae* for long time, several serious problems against* S. pneumoniae* therapy have occurred. One of them is the attenuation of the antibiotic effect. A number of antimicrobials that were previously useful in the treatment of acute bacterial sinusitis had MIC90s (Minimum Inhibitory Concentrations 90%) against intermediately resistant* S. pneumoniae* that were not achievable [2]. Furthermore, recently, multidrug-resistant* S. pneumoniae* has been increasing in Japan and worldwide [3]. As* S. pneumoniae* get antibacterial activity gradually, new anti-infective therapy against* S. pneumoniae* is desired.

Acute sinusitis is a common disease characterized by recurrent or persistent inflammation of the nasal and the paranasal sinus mucosa. It can be defined as an inflammation of paranasal sinus mucosa less than 3 weeks in duration [4]. As most acute cases result from infection, there is evidence that, in acute bacterial and viral sinusitis, proinflammatory cytokines play a dominant role in initiating and maintaining the inflammation, which is characterized by neutrophil tissue infiltration [5].

Traditional Chinese medicine (TCM) is one of the most popular alternative, complementary therapies worldwide [6]. It is becoming a popular alternative in otorhinolaryngology, where its use in the treatment of sinusitis, tinnitus, deafness, and Meniere's disease is growing [7]. In Japan, Kampo medicine, which had been developed from ancient Chinese medicine, is recognized as an effective alternative medicine against several diseases [8, 9]. Shin'iseihaito (Xinyiqingfeitang) is a formula in both traditional Japanese Kampo medicine and Chinese medicine, which is used for the treatment of upper respiratory tract diseases, especially sinusitis, in Japan [10, 11]. In Taiwan, the clinical investigation of the characteristics of adjunctive traditional Chinese medicine use in patients with chronic rhinosinusitis demonstrated that the most common Chinese herbal formula used was Xinyiqingfeitang [12]. In our previous basic studies, we investigated the antimicrobial effect of Shin'iseihaito extract (SSHT) in a pneumococcus-infected model [13], the antibacterial activity of SSHT against* S. pneumoniae* in vitro [14], and the preventive effect of SSHT in an ovalbumin-induced allergic rhinitis model [15]. However, experimental evidences on the use of SSHT for the treatment of bacterial sinusitis such as* S. pneumoniae* infection have not been elucidated yet.

In the present study, we evaluated the efficacy of SSHT against* S. pneumoniae* using a nasal infection murine model like sinusitis. Furthermore, we also evaluated the phagocytosis and chemotaxis effect of murine macrophage from SSHT-treated mouse.

## 2. Materials and Methods

### 2.1. Bacterial Strains

*Streptococcus pneumoniae (S. pneumoniae)* (ATCC49619) (American Type Culture Collection, Rockville, MD, USA) was used in this study. ATCC49619 (cps type: 19A) was isolated from sputum of a 75-year-old male. A fresh colony was inoculated overnight on TSAII sheep blood agar (Nihon Becton Dickinson, Tokyo, Japan) and cultured for 16 hours at 37°C under 5% CO_2_. The bacteria were harvested by centrifugation and resuspended in sterile phosphate-buffered saline (0.15 M, pH 7.2, PBS). Bacterial density was determined by measuring the absorbance at 600 nm (*A*_600_). The bacterial suspension was then diluted with PBS to 10^9^ CFU (colony forming unit)/mL using a standard growth curve to relate measured *A*_600_ to bacterial concentration.

### 2.2. Crude Drugs

Shin'iseihaito (Xinyiqingfeitang) (daily dose for human) consists of 3.0 g of the rhizome of* Anemarrhena asphodeloides* (AA), 1.0 g of the rhizome of* Cimicifuga heracleifolia* (CH), 2.0 g of the leaf of* Eriobotrya japonica *(EJ), 5.0 g of* Gypsum fibrosum *(GF), 3.0 g of the fruit of* Gardenia jasminoides *(GJ), 3.0 g of the bulb of* Lilium lancifolium *(LL), 3.0 g of the flower of* Magnolia salicifolia *(MS), 5.0 g of the tuber of* Ophiopogon japonicas *(OJ), and 3.0 g of the root of* Scutellaria baicalensis *(SB). These crude drugs were purchased from Daikoshoyaku (Nagoya, Japan) or Tsumura (Tokyo, Japan) and were standardized by Japanese Pharmacopoeia 17th Edition [16]. The mixture of above crude drugs was boiled in 20 times the weight of water for 30 min and filtered. The decoction was lyophilized to yield powdered extract (SSHT, 7.1 g for daily human dose). Fingerprint pattern of this SSHT was shown in our previous study [14]. SSHT was suspended in distilled water to prepare the stock solution at a concentration of 0.1 g/mL and kept in −20°C until use.

### 2.3. Murine Model of Nasal Infection

**Figure 1:**
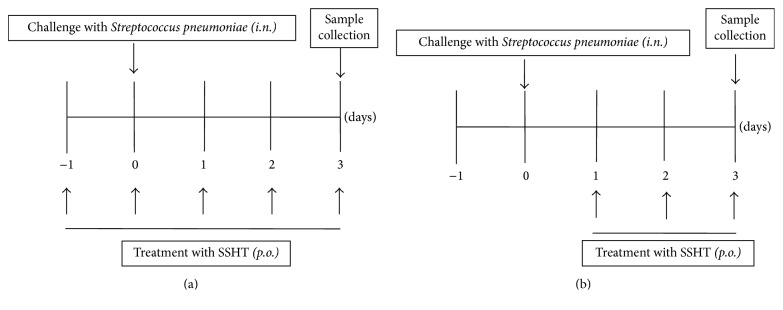
Protocols for the prophylactic (a) and therapeutic (b) experiments of* S. pneumoniae-*induced murine sinusitis model. In infected group, 1 × 10^7^ CFU bacteria were injected into both nostrils of mice using a 29-gauge needle at day 0. In the SSHT-treated group, mice were gavaged with Shin'iseihaito extract (SSHT) (2.9 g/kg body weight (bw))/day.

The ability of prophylactic effect of* S. pneumoniae* to cause sinusitis in mice after nasal inoculation was assessed using a procedure as follows [17]. In brief,* S. pneumoniae* was harvested after 16-hour growth on TSAII sheep blood agar and was mixed in 1 mL of PBS and then centrifuged at 2,000 ×g for 2 min. The pellets were diluted in 100 *μ*L PBS to 1 × 10^8^ CFU and then inoculated into the both nostrils of inbred 5-week-old male ddY mice (Japan SLC, Shizuoka, Japan) using a 29-gauge needle. The number of CFU inoculated was verified for each experiment by plating the bacteria on TSAII sheep blood agar and counting CFU. Mice were observed daily. In the SSHT-treated group, mice were gavaged with SSHT (0.69, 1.39, or 2.9 g/kg body weight (bw)) on days −1, 0, 1, 2, and 3 after* S. pneumoniae* inoculation (Figure 1(a)). We also performed another protocol to clarify the therapeutic effects of SSHT. In SSHT-treated group, mice were gavaged with SSHT (2.9 g/kg bw) on days 1, 2, and 3 after* S. pneumoniae *inoculation (Figure 1(b)). Mice in SSHT-untreated group for both protocols were given an equal volume of PBS instead of SSHT and were infected using the same method. The mice in control group for prophylactic protocol were given PBS without infection.

### 2.4. Nasal Lavage Cultures

The procedure of nasal cultures was described elsewhere [17]. In brief, the mice were sacrificed by CO_2_ inhalation. After that, the external nares, oral cavity, and head were disinfected with a moist alcohol swab and allowed to dry. Nasal lavage was performed with 200 *μ*L of PBS. The recovered fluid was then serially diluted, and 10 *μ*L of each dilution was plated onto TSAII sheep blood agar plates. The plates were incubated for 24 h, and then colonies of* S. pneumoniae *were counted. The results were quantified as the number of CFU/mL.

### 2.5. Histological Preparation

The histological preparation was assessed using a procedure described elsewhere [18]. Briefly, the head of mouse was stripped of eyes, skin, and muscle under low magnification, and the mandibles and tongue were discarded. After the head was then soaked in fixative overnight, it was decalcified and was trimmed with a fresh razor blade. The resulting blocks were embedded in paraffin sectioned anterior to posterior at 5 *μ*m thickness and stained with hematoxylin-eosin (H&E) method.

### 2.6. Scanning Electron Microscopic Analysis

Scanning electron microscopic preparation was performed according to what was described elsewhere [19]. Briefly, the head of mouse was stripped of eyes, skin, and muscle under low magnification, and the mandibles and tongue were discarded. Tissue was taken only if the debridement of the tissue was necessary during the surgical treatment. The tissue samples were immediately placed in 2.5% glutaraldehyde (Nisshin EM, Tokyo, Japan) (prepared in 0.1 M phosphate buffer, pH 7.4) for 24 h at 4°C as a prefixation step. They were then rinsed twice with 0.1 M phosphate buffer (pH 7.4), postfixed using 2% osmium tetroxide (Nisshin EM, Tokyo, Japan) for 2 h at room temperature, and finally rinsed with distilled water. Next, the specimens were dehydrated using graduated concentrations of ethyl alcohol (30%, 50%, 70%, 90%, 95%, and 100%) for 30 min, each followed by absolute alcohol for 30 min. The specimen was dried using the critical point dryer CPD300 (Leica, Wetzlar, Germany). For mounting, carbon conductive paint was used; for specimens, osmium coating with Osmium Coater (NL-OPC-AJ, Filgen, Nagoya, Japan) was used. Finally, each specimen was examined using a scanning electron microscope (SEM) (Hitachi High-Technologies S-4800). Several areas of each sample were systematically scanned.

### 2.7. Ex Vivo Isolation of SSHT-Treated Macrophage Cells

The procedure of ex vivo isolation of macrophage cells collected from SSHT-treated mice was modified as previous report [20]. In the SSHT-treated group, mice were gavaged with SSHT (2.9 g/kg body weight (bw)) for 4 days. Mice in the untreated group were given an equal volume of PBS. Then, peritoneal macrophage cells were harvested by lavage with 6 mL of cold sterilized PBS from the peritoneal cavities. The cells were washed twice and suspended in culture medium (RPMI1640 (Wako Pure Chemical Industry, Osaka, Japan) supplemented with 5% fetal bovine serum (FBS, Sigma-Aldrich, St. Louis, MO, USA)) to give 10^6^ cells/mL. 100 *μ*L of the cell suspension was dispensed into 1.5 mL tube and incubated for 2 h at 37°C in 5% CO_2_. The culture medium was centrifuged and removed gently by aspiration and changed to fresh culture medium. All cells were suspended in the culture medium, plated onto 24-well culture plate at dose of 1 × 10^5^ cells per well (final volume, 0.1 mL), and cultured in a humidified chamber at 37°C with 5% CO_2_ atmosphere for 24 h.

### 2.8. Determination of Macrophage Proliferative Response

At 20 h before the end of the macrophage culture, ^3^H-thymidine (2.0 Ci/mmol; PerkinElmer, MA, USA) was added to the medium in the wells. When the culture was finished, the cells were adsorbed on 0.45 *μ*m membrane filters (Advantech Japan, Tokyo, Japan), washed with distilled water, and then dried. The filters were transferred to vials filled with liquid scintillator cocktail, and the radioactivity was measured with a liquid scintillation counter (LSC-6100, Hitachi Aloka Medical, Tokyo, Japan). Results are given as D.P.M. ± SD of triplicate samples.

### 2.9. Macrophage Phagocytic Assay

The procedure of macrophage phagocytic assay was modified as previous report [20]. Mouse macrophage (2 × 10^6^ cells in 100 *μ*L) and* S. pneumoniae *(10^4^ CFU in 100 *μ*L) that was opsonized with mouse serum for 30 min were incubated at 37°C for 1 h with shaking on a rotator in 5% CO_2_ atmosphere. Then, the aliquots were plated on TSAII sheep blood agar to determine the number of CFU after 0, 30, 60, 90, and 120 min. Data were expressed as bacterial CFU counts at each time.

### 2.10. Cytokine Production Analysis by ELISA

Peritoneal macrophages were cultured in a 24-well (1 × 10^5^/well) plate in RPMI1640 containing 5% FBS, 100 U/mL penicillin, and 100 *μ*g/mL streptomycin (GE Healthcare, Chicago, IL, USA) and incubated for 24 h. Then, the medium was collected, and the concentrations of tumor necrosis factor-*α* (TNF-*α*), interleukin-1*β* (IL-1*β*), IL-6, and monocyte chemotactic protein-1 (MCP-1) were measured using enzyme-linked immunosorbent assay (ELISA) kits (BioLegend, San Diego, CA, USA).

### 2.11. Macrophage Chemotaxis Assay

Chemotaxis of peritoneal macrophages was assayed using 24-well chemotactic chambers (pore size, 5 *μ*m; Costar, ME, USA). The Zymosan-activated serum was prepared by adding 10 mg of Zymosan (Sigma) to 1 mL of FBS and incubation at 37°C for 30 min. After incubation, the serum was centrifuged at 1,000 ×g for 10 min and diluted with PBS to give a 20% solution [21]. The upper chamber compartment was loaded with 100 *μ*L mouse peritoneal macrophage suspension (1 × 10^6^ cells). The lower compartment was filled with 900 *μ*L RPMI1640 medium with both 2% FBS and Zymosan-activated serum. Following 24 h incubation at 37°C with 5% CO_2_ atmosphere, the lower surface of chambers was stained with Giemsa staining, and the number of cells that had migrated to the lower surface of chamber was counted by fluorescence microscope (Eclipse E800, Nikon Instruments Co., Ltd., Tokyo). Data was determined as the mean of six random high-power fields per well. Each assay was performed at least three times. The chemotactic index was the ratio of the number of cells that had migrated in response to Zymosan-activated serum to the unmigrated number of cells as described before [22].

### 2.12. Statistical Analysis

The statistical analysis was conducted using an unpaired Student's* t*-test for two groups and Tukey's multiple comparison test for the differences among multiple groups or two-way ANOVA followed by Tukey's test for time course study of 3 groups. *p* values less than 0.05 were considered statistically significant.

## 3. Results

### 3.1. Murine Nasal Infection Model

**Figure 2:**
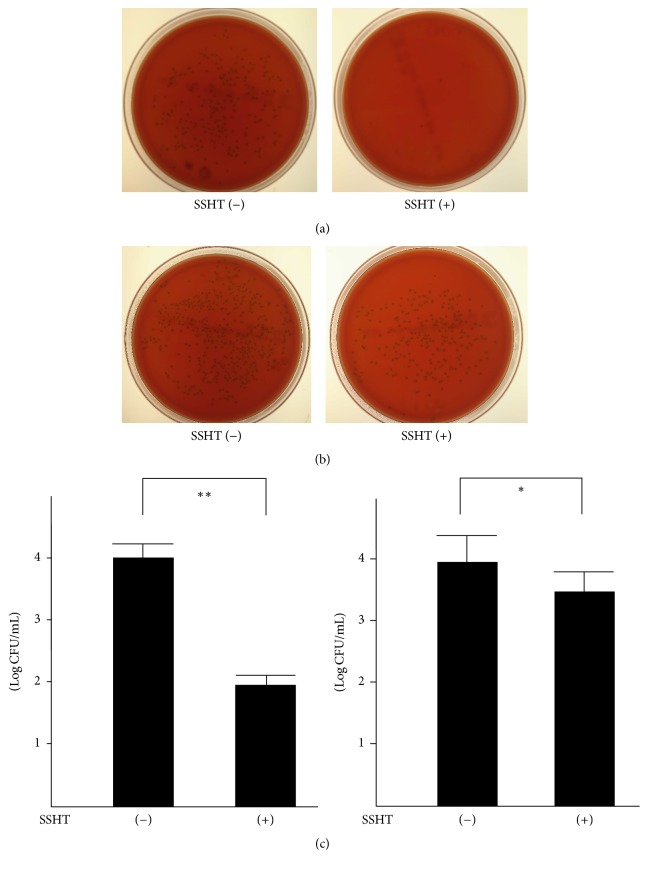
The colonies of* S. pneumoniae* in SSHT-treated and untreated murine nose in (a) prophylactic and (b) therapeutic experiments. The nasal fluids were inoculated on TSAII sheep blood agar and incubated for 24 h. (c) Comparison of colony count between SSHT-treated and untreated mice in prophylactic (left) and therapeutic (right) model. Dosage of SSHT was 2.9 g/kg bw/day. Data represent the mean ± SD (*n* = 6). ^*∗*^*p* < 0.05 and ^*∗∗*^*p* < 0.01 by Student's* t*-test. SSHT: Shin'iseihaito extract.

**Figure 3:**
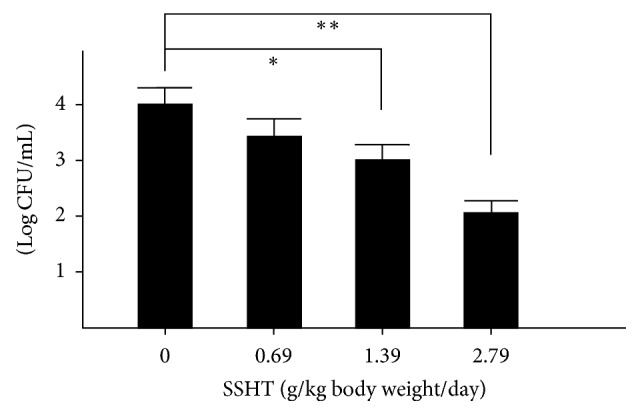
Dose-dependent effect of SSHT on nasal* S. pneumoniae* infection. Each dosage of SSHT was treated in the prophylactic protocol. Data represent the mean ± SD (*n* = 6). ^*∗*^*p* < 0.05 and ^*∗∗*^*p* < 0.01 versus untreated group by Tukey's multiple comparison test. SSHT: Shin'iseihaito extract.

**Figure 4:**
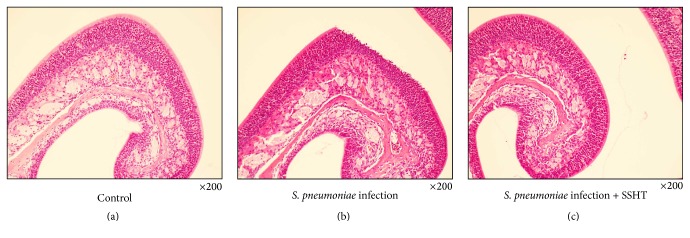
Histopathological analysis of nose. Histological analysis represented H&E staining. (a) Control; (b)* S. pneumoniae*-infected SSHT-untreated mice; (c)* S. pneumoniae*-infected SSHT-treated mice. SSHT (2.9 g/kg bw/day) was treated in the prophylactic protocol. SSHT: Shin'iseihaito extract.

**Figure 5:**
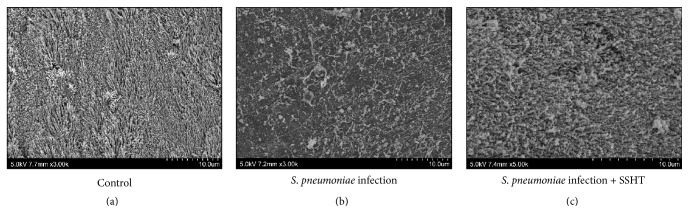
Scanning electron microscopic analysis of nose. (a) Control; (b)* S. pneumoniae*-infected SSHT-untreated mice; (c)* S. pneumoniae-*infected SSHT-treated mice. SSHT (2.9 g/kg bw/day) was treated in the prophylactic protocol. SSHT: Shin'iseihaito extract.

First of all, we tried to assess whether SSHT would provide the host with a prophylactic or therapeutic effect against* S. pneumoniae* nasal infection. Four days after nostril infection of* S. pneumoniae*, we evaluated the bacterial colony counts in murine nose. In prophylactic protocol, the CFU count in the nose of SSHT-treated mouse (2.9 g/kg bw/day, 20 times the human daily dosage) was significantly lower than that in the nose of SSHT-untreated mouse (*p* < 0.01) (Figure 2(a)). In therapeutic protocol, the CFU count in the nose of SSHT-treated mouse was also significantly lower than that in the nose of SSHT-untreated mouse (*p* < 0.05) (Figure 2(b)); however, the prophylactic administration was more effective than the therapeutic administration in SSHT treatment (Figure 2(c)). In prophylactic protocol, SSHT dose-dependently reduced the colony formation of* S. pneumoniae *in the nose of mice, and 10 times the human daily dosage of SSHT exhibited statistically significant decrease (Figure 3). Histopathological analysis showed the destruction of epithelial nasal mucosa in* S. pneumoniae*-infected mice, and we confirmed the improvement of epithelial nasal mucosal disruption in SSHT-treated mice in prophylactic protocol (Figure 4). Scanning electron microscopic (SEM) analysis demonstrated the extensive destruction of ciliated epithelial cells in* S. pneumoniae*-infected mice, and incomplete recovery of ciliated epithelial cells was found in SSHT-treated mice in prophylactic protocol (Figure 5).

### 3.2. Phagocytic Activity of Peritoneal Macrophage

**Figure 6:**
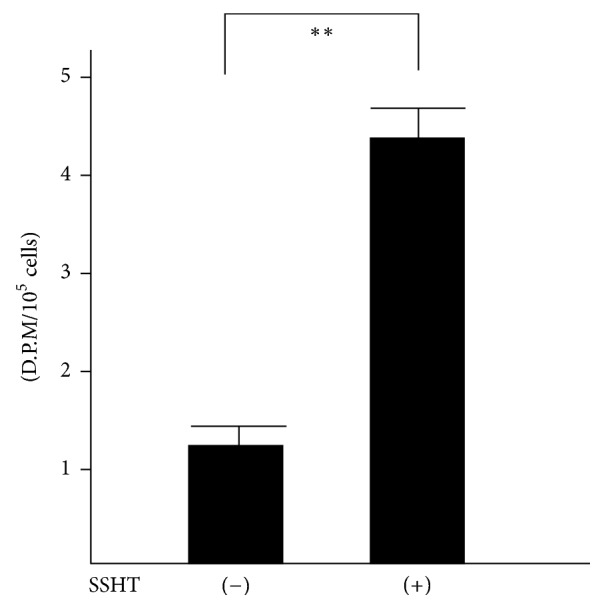
^3^H-thymidine-uptake assay in peritoneal macrophage. SSHT (2.9 g/kg bw/day) was gavaged for 4 days, and peritoneal macrophages were collected. Data represent the mean ± SD (*n* = 6). ^*∗∗*^*p* < 0.01 by Student's* t*-test. SSHT: Shin'iseihaito extract.

**Figure 7:**
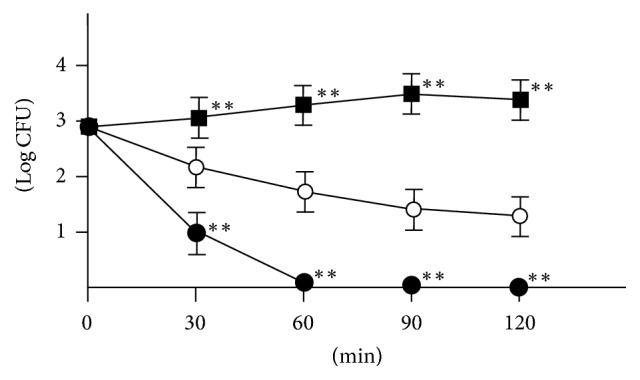
Macrophage phagocytosis assay in peritoneal macrophage. Black square, control; open circle, macrophages from untreated mice; close circle, macrophages from SSHT-treated mice. SSHT (2.9 g/kg bw/day) was gavaged for 4 days, and peritoneal macrophages were collected. Data represent the mean ± SD (*n* = 6). ^*∗∗*^*p* < 0.01 versus untreated group by two-way ANOVA followed by Tukey's test. SSHT: Shin'iseihaito extract.

We next focused on macrophage activity, because macrophage plays a major role in phagocytic and chemotactic activity in murine bacterial infection model. To determine whether peritoneal macrophages from SSHT-treated mice showed elevated activities, we performed ^3^H-thymidine uptake analysis. As shown in Figure 6, the uptake of ^3^H-thymidine into peritoneal macrophages collected from SSHT-treated mice was significantly higher than that from untreated mice (*p* < 0.01). As we inferred that SSHT might activate the peritoneal macrophages, we performed their phagocytic assays on* S. pneumoniae*. As shown in Figure 7, macrophages from untreated mice killed approximately 90% of the* S. pneumoniae* inoculum after 30-minute incubation at 37°C, and the macrophages from SSHT-treated mice killed more than 99% of* S. pneumoniae* inoculum. We did not find any bacterial colony at 60-minute incubation under SSHT treatment. Significant differences were observed in* S. pneumoniae *CFU reduction between SSHT-treated and untreated mice (*p* < 0.01).

### 3.3. Cytokine Expression of Peritoneal Macrophage

**Figure 8:**
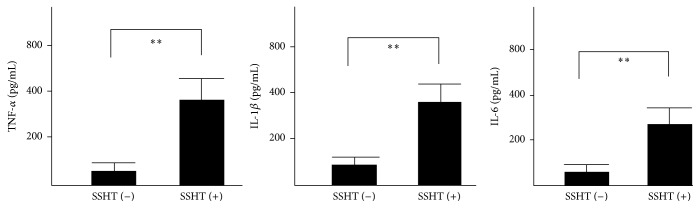
Cytokine levels in culture medium of macrophage. TNF-*α*, IL-1*β*, and IL-6 were measured by ELISA. SSHT (2.9 g/kg bw/day) was gavaged for 4 days, and peritoneal macrophages were collected. Data represent the mean ± SD (*n* = 6). ^*∗∗*^*p* < 0.01 by Student's* t*-test. SSHT: Shin'iseihaito extract.

We examined the cytokine expression from peritoneal macrophages induced by SSHT treatment. After 24 h incubation of macrophages, the levels of TNF-*α*, IL-1*β*, and IL-6 in culture medium of peritoneal macrophages were measured by ELISA. Our results showed that the levels of these cytokines were significantly upregulated in mice treated with SSHT compared with those in untreated mice (*p* < 0.01, resp., Figure 8).

### 3.4. Effect of Chemotaxis Activity by Peritoneal Macrophage

**Figure 9:**
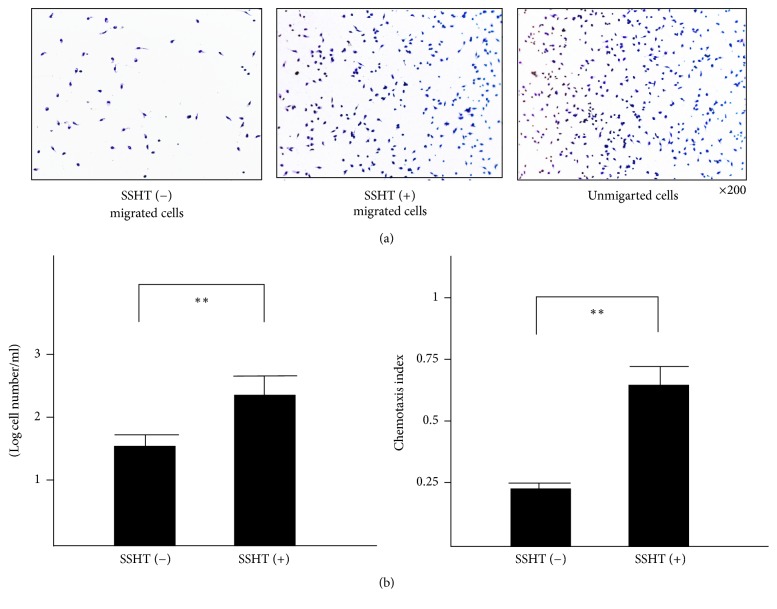
Ex vivo macrophage chemotaxis analysis. Chemotaxis of peritoneal macrophages was assayed using 24-well chemotactic chambers. (a) Left panel, migrated macrophages from SSHT-untreated mice; middle panel, migrated macrophages from SSHT-treated mice; right panel, unmigrated macrophage. (b) Comparison of chemotaxis assay between SSHT treatment and control. Left panel, the number of chemotaxis macrophages; right panel, chemotaxis index. SSHT (2.9 g/kg bw/day) was gavaged for 4 days, and peritoneal macrophages were collected. Data shown represent the mean ± SD (*n* = 6).  ^*∗∗*^*p* < 0.01 by Student's* t*-test. SSHT: Shin'iseihaito extract.

To determine whether peritoneal macrophages from SSHT-treated mice upregulated the chemotaxis activity, we performed the chemotaxis assays on* S. pneumoniae*. As shown in Figure 9, the peritoneal macrophages from SSHT-treated mice migrated more than those from untreated mice. We also found significant differences of migrating cell number and chemotaxis index between macrophages from SSHT-treated and untreated mice (*p* < 0.01).

### 3.5. Migration Associated Cytokine Expression of Peritoneal Macrophage

**Figure 10:**
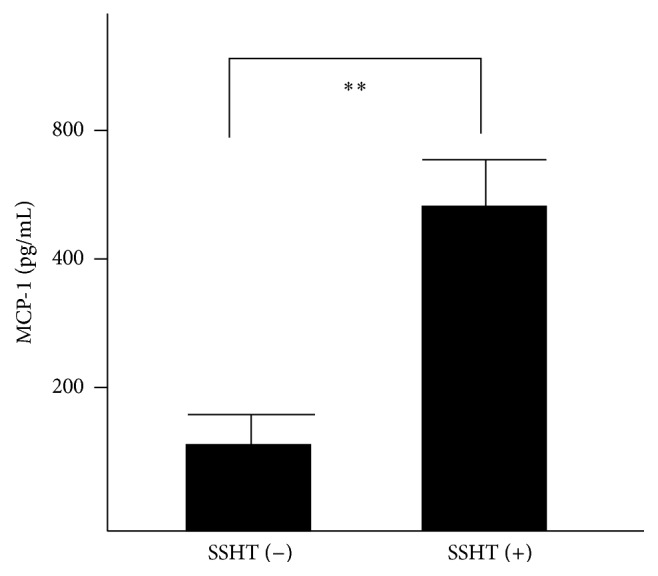
Chemotaxis cytokine production from macrophage. SSHT (2.9 g/kg bw/day) was gavaged for 4 days, and peritoneal macrophages were collected. MCP-1 was measured by ELISA. Data represent the mean ± SD (*n* = 6). ^*∗∗*^*p* < 0.01 by Student's* t*-test. SSHT: Shin'iseihaito extract.

Finally, we examined the migration associated cytokine expression from peritoneal macrophage induced by SSHT. After 24 h incubation of macrophage, MCP-1 levels in culture medium of peritoneal macrophage were measured by ELISA (Figure 10). The result showed that MCP-1 was significantly upregulated under SSHT treatment (*p* < 0.01).

## 4. Discussion

To our knowledge, this is the first experimental study in which SSHT would be effective in* S. pneumoniae*-caused murine sinusitis model.

After nasal* S. pneumoniae* infection, SSHT-treated mice showed reduction of* S. pneumoniae* colonization in murine nose, improvement of destruction of epidermal mucosa, macrophage phagocytic activity, upregulation of peritoneal macrophage producing cytokines including TNF-*α*, IL-1*β*, IL-6, and MCP-1, and macrophage chemotaxis activity. These results suggest that SSHT can play an important role in protection against* S. pneumoniae* in mouse sinusitis model.

At first, we tried to clarify the mechanism of this prophylactic effect of SSHT. Although we revealed the direct in vitro antibacterial effect of SSHT on* S. pneumoniae* in previous study [14], the protective effect provided by SSHT would not be due to direct antibacterial effect of SSHT, because the administration of SSHT was oral and all constituents of SSHT would not be absorbed from the intestine and appeared at nose as their original form. In addition, we next tried to clarify whether SSHT would provide the protection if given only after bacterial infection. Even though we found the significant recovery of colonization rate in direct SSHT administration only after* S. pneumoniae* infection, the therapeutic effect was less than the prophylactic effect in SSHT treatment. Our data imply that SSHT may have potent preventive effect on the infection of* S. pneumoniae* and may be useful for both prophylactic and therapeutic administration for* S. pneumoniae* sinusitis.

Our histopathological results showed the improvement of epithelial nasal mucosal disruption in SSHT-treated mouse in comparison to the epithelial nasal mucosa in* S. pneumoniae*-infected mouse. This is consistent with the previous report demonstrating that experimental sinus infection in the rabbit model leads to marked destruction of ciliated epithelial cells [23].

The phagocytic activity of murine peritoneal macrophage was enhanced in the SSHT-treated group. As a result of systemic immunomodulatory action by the orally administered SSHT extract, activation of phagocytic ability of macrophages as a local immunomodulatory effect occurs in the nasal cavity. It is common for macrophages circulating in the blood to aggregate with migratory cytokines to inflamed tissue sites [24]. Peritoneal macrophages can also be considered as a type of systemic macrophages infiltrated from capillaries such as the abdominal wall. Therefore, peritoneal macrophages can be used as functional analysis of macrophages in local areas such as nasal cavities. Macrophages collected from SSHT-treated mice showed significantly higher TNF-*α*, IL-1*β*, IL-6, and MCP-1 production than those from untreated mice. In this study, we focused on inflammatory cytokines (TNF-*α*, IL-1*β*, and IL-6) and migratory cytokines (MCP-1). Those inflammatory cytokines activate the phagocytosis of macrophage. Migratory cytokine also activates the migration of macrophage. Bacterial infection induces inflammatory cytokines and causes inflammation. After migratory cytokines of leukocytes are induced for phagocytosis of bacteria, leukocytes assemble in the lesion area. Although the relationship between inflammatory cytokine and* S. pneumoniae*-caused sinusitis has been unclear, previous report showed that* S. pneumoniae *induced inflammatory cytokine from bronchoalveolar lavage fluid sample in mouse pneumonia model [25, 26]. Another report also revealed that* S. pneumoniae* induced MCP-1 in human epithelial cells [27]. Our results were also consistent with previous reports. We suggested that SSHT-induced cytokine production augments bactericidal activity including phagocytic activity which in turn causes bacterial elimination. Our results also demonstrated that SSHT upregulated the chemotaxis activity of macrophages which played important roles to prevent bacterial infection to the host. Our data also potentially postulate that SSHT may be useful in the treatment of multidrug-resistant* S. pneumoniae*, because SSHT is not per se an antibiotic but it is an immunomodulator. Although SSHT has antibacterial effect against* S. pneumoniae* [14], the occurrence of SSHT-resistant* S. pneumoniae* in future seems to be impossible, and we speculate that SSHT may be efficacious when used against new strains of antibiotic-resistant* S. pneumoniae *strain. We also investigated the effect of SSHT against other cps-type* S. pneumoniae* isolates and confirmed that SSHT had the biofilm inhibitory effect on those isolates in vitro. The relationship between specific cps-type isolates and effects of SSHT may not appear. Our results imply that SSHT may also affect the host's immune system with regard to* S. pneumoniae* infection.

We investigated the function of murine peritoneal macrophage by ex vivo analysis. The ex vivo analysis has the great advantage to more closely reflect in vivo conditions compared to the results obtained using cell culture systems. Indeed, this ex vivo method will be valuable in examining the effects of SSHT therapeutic strategies on macrophage, as it might better reflect in vivo conditions compared to the experiments using direct treatment to cultured tissue or cells, where artefacts are often observed.

Juzentaihoto, one of Kampo formulae, upregulated the macrophage phagocytosis [28] and enhanced IL-12 production by modulating Toll-like receptor 4 signaling pathways in murine peritoneal exudate macrophages [29]. Ingestion of Kakkonto, another Kampo formula, not only increased the body temperature but also enhanced the phagocytic activity of macrophages, which was an in vivo defence mechanism [30]. Although Kampo medicines have immunomodulating effect such as the activation of macrophage [20], these mechanisms in detail have been unknown. Kampo medicines are generally composed of several crude drug components, and the interaction of them may enhance the effect of drugs. Since Shin'iseihaito does not contain the same crude drug components as Juzentaihoto or Kakkonto, we cannot deduce the relevance of the immune activation effect between these three drugs, and the immunomodulating crude drugs might newly be found among the components of Shin'iseihaito. As further investigation from these perspectives is needed, SSHT may have pronounced immunomodulating effects. And further investigations involving human subjects about* S. pneumoniae* and other bacterial infections using SSHT are also desired.

In summary, Shin'iseihaito is significantly effective for the treatment of* S. pneumoniae* sinusitis in murine model. We suggest Shin'iseihaito as the therapeutic candidate for novel effective therapy on human sinusitis caused by* S. pneumoniae*.

## References

[1] Kadioglu A., Weiser J. N., Paton J. C., Andrew P. W. (2008). The role of Streptococcus pneumoniae virulence factors in host respiratory colonization and disease. *Nature Reviews Microbiology*.

[2] Doern G. V., Brueggemann A., Holley H. P., Rauch A. M. (1996). Antimicrobial resistance of *Streptococcus pneumoniae* recovered from outpatients in the United States during the winter months of 1994 to 1995: results of a 30-center national surveillance study. *Antimicrobial Agents and Chemotherapy*.

[3] Minami M., Sakakibara R., Imura T., Morita H., Kanemaki N., Ohta M. (2015). Relationship between clinical characteristics and multidrug-resistant patterns of *Streptococcus pneumoniae* in Japan. *International Journal of Current Research in Biosciences and Plant Biology*.

[4] Benninger M. S., Ferguson B. J., Hadley J. A. (2003). Adult chronic rhinosinusitis: definitions, diagnosis, epidemiology, and pathophysiology. *Otolaryngology - Head and Neck Surgery*.

[5] Min Y.-G., Lee K. S. (2000). The role of cytokines in rhinosinusitis. *Journal of Korean Medical Science*.

[6] Park H.-L., Lee H.-S., Shin B.-C. (2012). Traditional medicine in China, Korea, and Japan: a brief introduction and comparison. *Evidence-Based Complementary and Alternative Medicine*.

[7] Yap L., Pothula V. B., Warner J., Akhtar S., Yates E. (2009). The root and development of otorhinolaryngology in traditional Chinese medicine. *European Archives of Oto-Rhino-Laryngology*.

[8] Uezono Y., Miyano K., Sudo Y., Suzuki M., Shiraishi S., Terawaki K. (2012). A review of traditional Japanese medicines and their potential mechanism of action. *Current Pharmaceutical Design*.

[9] Motoo Y., Seki T., Tsutani K. (2011). Traditional Japanese medicine, Kampo: its history and current status. *Chinese Journal of Integrative Medicine*.

[10] Majima Y., Sakakura Y., Hamaguchi F., Murai S. (1992). Shin'i-Seihai-To (TJ-104) treatment of chronic sinusitis. *Practica Oto-Rhino-Laryngologica*.

[11] Kato M., Hattori T., Beppu R., Kitamura M., Yanagita N. (1994). Effectiveness of Shin'i-Seihai-To for Sinusitis with Polyp.. *Jibirinsyo*.

[12] Yen H.-R., Sun M.-F., Lin C.-L., Sung F.-C., Wang C.-C., Liang K.-L. (2015). Adjunctive traditional Chinese medicine therapy for patients with chronic rhinosinusitis: a population-based study. *International Forum of Allergy and Rhinology*.

[13] Minami M., Konishi T., Jiang Z., Arai T., Makino T. (2015). Effect of shin'iseihaito on lung colonization of pneumococcus in murine model. *African Journal of Traditional, Complementary and Alternative Medicines*.

[14] Konishi T., Minami M., Jiang Z., Arai T., Makino T. (2016). Antibacterial activity of Shin'iseihaito (Xin Yi Qing Fei Tang) against streptococcus pneumoniae. *Pharmacognosy Journal*.

[15] Minami M., Konishi T., Jiang Z., Arai T., Makino T. (2016). Effect of Shin'iseihaito on murine allergic reaction induced by nasal sensitization. *Journal of Traditional and Complementary Medicine*.

[16] Ministry of Health Japan The Japanese Pharmacopoeia, Seventeenth Edition. http://jpdb.nihs.go.jp/jp17e/.

[17] Blair C., Naclerio R. M., Yu X., Thompson K., Sperling A. (2005). Role of type 1 T helper cells in the resolution of acute *Streptococcus pneumoniae* sinusitis: a mouse model. *Journal of Infectious Diseases*.

[18] Bomer K., Brichta A., Baroody F., Boonlayangoor S., Li X., Naclerio R. M. (1998). A mouse model of acute bacterial rhinosinusitis. *Archives of Otolaryngology - Head and Neck Surgery*.

[19] Berger G., Kattan A., Bernheim J., Ophir D., Finkelstein Y. (2000). Acute sinusitis: a histopathological and immunohistochemical study. *Laryngoscope*.

[20] Minami M., Ichikawa M., Hata N., Hasegawa T. (2011). Protective effect of hainosankyuto, a traditional japanese medicine, on streptococcus pyogenes infection in murine model. *PLoS ONE*.

[21] Anjo S., Kondo Y., Ishibashi Y., Arai T. (1992). Effect of antibiotics on chemotaxis of human neutrophils. *Chemotherapy*.

[22] Yuan D.-P., Gu L., Long J. (2014). Shikonin reduces endometriosis by inhibiting RANTES secretion and mononuclear macrophage chemotaxis. *Experimental and Therapeutic Medicine*.

[23] Fukami M., Norlander T., Stierna P., Westrin K. M., Carlsöö B., Nord C. E. (1993). Mucosal pathology of the nose and sinuses: a study in experimental maxillary sinusitis in rabbits induced by *Streptococcus pneumoniae*, *Bacteroides fragilis*, and *Staphylococcus Aureus*. *American Journal of Rhinology*.

[24] Albegger K. W. (1976). Structure and function of the mononuclear phagocytic system (MPS) in chronic rhinosinusitis: a light and electron microscopic investigation. *Archives of Oto-Rhino-Laryngology*.

[25] McKenzie C. W., Klonoski J. M., Maier T. (2013). Enhanced response to pulmonary Streptococcus pneumoniae infection is associated with primary ciliary dyskinesia in mice lacking Pcdp1 and Spef2. *Cilia*.

[26] Peñaloza H. F., Nieto P. A., Muñoz-Durango N. (2015). Interleukin-10 plays a key role in the modulation of neutrophils recruitment and lung inflammation during infection by Streptococcus pneumoniae. *Immunology*.

[27] Shin H.-S., Yoo I.-H., Kim Y.-J. (2010). MKP1 regulates the induction of MCP1 by Streptococcus pneumoniae pneumolysin in human epithelial cells. *Molecules and Cells*.

[28] Liu H., Wang J., Sekiyama A., Tabira T. (2008). Juzen-taiho-to, an herbal medicine, activates and enhances phagocytosis in microglia/macrophages. *Tohoku Journal of Experimental Medicine*.

[29] Chino A., Sakurai H., Choo M.-K. (2005). Juzentaihoto, a Kampo medicine, enhances IL-12 production by modulating Toll-like receptor 4 signaling pathways in murine peritoneal exudate macrophages. *International Immunopharmacology*.

[30] Muraoka K., Yoshida S., Hasegawa K. (2004). A pharmacologic study on the mechanism of action of Kakkon-to: body temperature elevation and phagocytic activation of macrophages in dogs. *Journal of Alternative and Complementary Medicine*.

